# Treatment of osteoporosis using a selective androgen receptor modulator ostarine in an orchiectomized rat model

**DOI:** 10.1007/s12020-023-03422-7

**Published:** 2023-06-28

**Authors:** K. O. Böker, M. Komrakova, L. Fahrendorff, B. R. Spelsberg, D. B. Hoffmann, A. F. Schilling, W. Lehmann, S. Taudien, S. Sehmisch

**Affiliations:** 1 Department of Trauma Surgery, Orthopaedics and Plastic Surgery, University Medical Center Goettingen, Goettingen, Germany; 2 Division of Infection Control and Infectious Diseases, University Medical Center Goettingen, 37075 Goettingen, Germany; 3 Department of Trauma Surgery, Hannover Medical School, University of Hannover, Carl-Neuberg-Str. 1, 30625 Hannover, Germany

**Keywords:** Osteoporosis, Orchiectomized (Orx) rat model, Selective androgen receptor modulator (SARM), Ostarine

## Abstract

**Purpose:**

The selective androgen receptor modulator ostarine has been shown to have advantageous effects on skeletal tissue properties, reducing muscle wasting and improving physical function in males. However, data on effects in male osteoporosis remain limited. In this study, the effects of ostarine on osteoporotic bone were evaluated in a rat model of male osteoporosis and compared with those of testosterone treatments.

**Methods:**

Eight-month-old male Sprague-Dawley rats were either non-orchiectomized to serve as a healthy control (Non-Orx, Group 1) or orchiectomized (Orx, Groups 2–6) and then grouped (*n* = 15/group): (1) Non-Orx, (2) Orx, (3) Ostarine Therapy, (4) Testosterone Therapy, (5) Ostarine Prophylaxis and (6) Testosterone Prophylaxis. Prophylaxis treatments started directly after orchiectomy and continued for 18 weeks, whereas Therapy treatments were initiated 12 weeks after Orx. Ostarine and Testosterone were applied orally at daily doses of 0.4 and 50 mg/kg body weight, respectively. The lumbar vertebral bodies and femora were analyzed using biomechanical, micro-CT, ashing, and gene expression analyses.

**Results:**

Ostarine Prophylaxis showed positive effects in preventing osteoporotic changes in cortical and trabecular bone (femoral trabecular density: 26.01 ± 9.1% vs. 20.75 ± 1.2% in Orx and in L4: 16.3 ± 7.3% vs 11.8 ± 2.9% in Orx); biomechanical parameters were not affected; prostate weight was increased (0.62 ± 0.13 g vs 0.18 ± 0.07 g in Orx). Ostarine Therapy increased solely the cortical density of the femur (1.25 ± 0.03 g/cm^3^ vs. 1.18 ± 0.04 g/cm^3^ in Orx); other bone parameters remained unaffected. Testosteron Prophylaxis positively influenced cortical density in femur (1.24 ± 0.05 g/cm^3^ vs. 1.18 ± 0.04 g/cm^3^ in Orx); Test. Therapy did not change any bony parameters.

**Conclusion:**

Ostarine Prophylaxis could be further investigated as a preventative treatment for male osteoporosis, but an androgenic effect on the prostate should be taken into consideration, and combination therapies with other anti-osteoporosis agents could be considered.

## Introduction

Osteoporosis is a widespread disease characterized by an imbalance between bone resorption and formation, leading to decreased bone mass. Because of the resulting instability of the bone tissue, fracture risk is increased in men and women. Postmenopausal osteoporosis in women is related to estrogen deficiency in menopause [1]. Thus far, osteoporosis research has focused mainly on treatments for women; however, hypogonadism and age-related hormone changes are associated with osteoporosis in men as well [2, 3]. According to Melton et al., 25% of men aged over 50 years will have an osteoporosis-related fracture [4], and this value will increase in the future [5, 6]. Among the 9 million fractures worldwide, 39% occur in male patients [7]. Furthermore, men show an increased mortality risk after fractures. Although the recognition of osteoporosis in men is increasing, there are only few medications for this disease, and they all have negative side effects [8–10].

In males, osteoporosis occurs primarily due to the reduced testicular testosterone production. Male osteoporosis is still an underdiagnosed and undertreated condition with severe consequences [8, 9, 11]. Testosterone therapy, commonly used to treat male hypogonadism and androgen deficiency of severe disease or aging, has been shown to result in significant improvements in muscle function, bone mineral density and bone healing [12]. Efficacy and mode of action of androgens are under discussion. Androgens may maintain trabecular bone by acting directly on osteocytes or indirectly by inhibiting osteoclastogenesis through interaction with osteoblast precursors, but have no direct effect on osteoclasts [13, 14]. Hypogonadism-induced bone loss can be treated with testosterone replacement therapy. However, the associated negative side effects limits the benefit, especially in older patients [11, 12].

Dissociating anabolic effects from androgenic activities is one approach to obtaining positive myoanabolic and osteoanabolic outcomes without side effects on the prostate in males or virilization in female patients. Steroidal compounds have failed to obtain desirable result, but some new non-steroidal compounds have shown, in vivo and in clinical trials, promising positive results [15–17]. One example is selective androgen receptor modulators (SARMs), which showed strongly dissociating anabolic and androgenic activities following androgen-receptor (AR) activation. These SARMs represent the androgenic counterpart of the selective estrogen receptor modulators (SERMs) in terms of tissue and action specificity [17].

Non-steroidal SARMs such as ostarine (GTx-24, MK-2866, or Enobosarm) lack steroid rings and subsequently possess selective effects on the skeletal system, reportedly showing only minimal effects on other androgen-dependent tissues [18, 19]. The advantage of SARMs is that their activity is directed towards the maintenance or enhancement of the anabolic effects on bone and muscle with a minimal androgenic effect on the growth of the prostate [20]. SARMs could provide therapeutic opportunities in a variety of diseases, including muscle wasting and osteoporosis in man, by maximizing the positive attributes of steroidal androgens while minimizing the negative side effects [20, 21]. Ostarine, developed by GTx Incorporated (Memphis, TN, USA) for the treatment of muscle wasting, showed positive effects on physical function and lean body mass in elderly men with tumor-induced muscle wasting [15, 16]. Most studies of ostarine treatments in male patients focus on muscle function, muscle wasting and physical function [15, 16], whereas data on SARM effects, particularly for ostarine in male osteoporosis, remain limited.

In previous studies, we demonstrated the beneficial effects of ostarine on bone mineral density and bone volume in an ovariectomized rat model of postmenopausal osteoporosis [18, 22]. Ostarine had also effects on osteoporotic bone healing in that ostarine prophylaxis treatment increased callus area and callus density while cortical density was decreased [22]. Therapeutic treatment with ostarine, on the other hand, affected bone healing negatively by reducing callus density and area and delaying osteotomy bridging [22].

In the present study, the effect of ostarine on bone tissue (lumbar vertebral body and femur) was evaluated in orchiectomized male rats and compared with testosterone treatments. Treatments were applied in the form of osteoporosis prophylaxis and therapy as well. Data from an animal model on body weight, food intake, prostate weight, and serum parameters have been published recently [22, 23].

## Material and methods

### General information

**Fig. 1:**
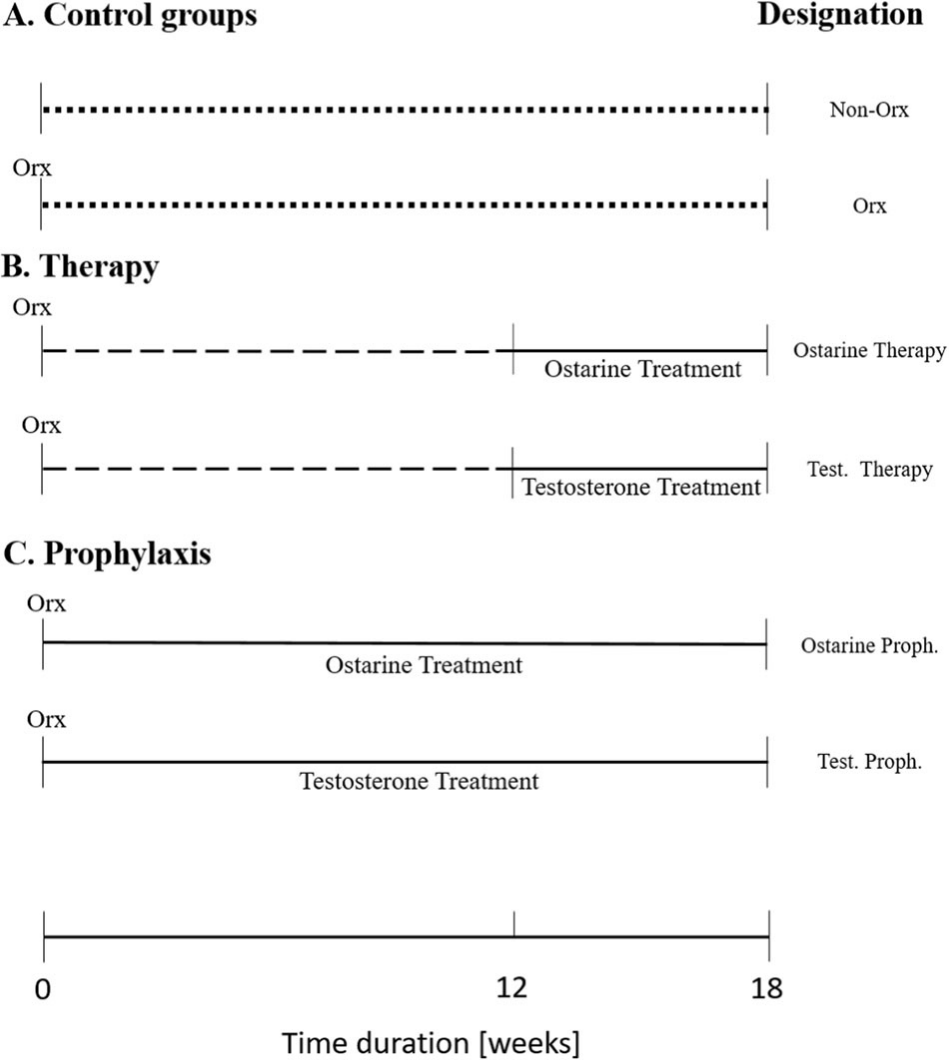
Study design. Eight-month-old Sprague-Dawley rats were assigned to 6 groups: (**A**) healthy, Non-Orx and osteoporotic Orx rats; (**B**) Orx rats treated either with ostarine or testosterone within week 12 and 18; (**C**) Orx rats treated with ostarine or testosterone within week 0 and 18. Eighteen weeks after Orx, samples were collected

The animal study protocol was approved by the local regional government (14/1396, Oldenburg, Germany) in accordance with German animal protection laws prior to performing the study. We used orchiectomized (Orx) male rats, which is the standard animal model for osteoporosis studies [24, 25]. The experiments were performed with 90 8-month-old male Sprague-Dawley rats (Janvier Labs, Le Genest-Saint-Isle, France). All rats were fed a soy-free diet throughout the experiment (ssniff Special Diet, Soest, Germany). One group (Non-Orx) received no therapy and no treatment (*n* = 15) and represented a healthy control group (Fig. 1). The remaining 75 rats were orchiectomized. No further treatment was performed in the Orx group, while ostarine or testosterone treatment was begun after orchiectomy (osteoporosis prophylaxis groups) or 12 weeks after the orchiectomy when osteoporotic changes in the bone occurred (osteoporosis therapy groups). A detailed study design is depicted in Fig. 1.

Ostarine and testosterone were added to the animals’ feed to achieve a daily dose of ostarine of 0.4 mg/kg body weight (BW) [18] and a daily dose of testosterone of 50 mg/kg BW [11, 26]. These concentrations showed the best balance between effect on bone and side effects on organs. Body weight and food intake were measured during the experiment on a weekly basis. The daily dosage calculated at the end of the experiment was averaged over the treatment weeks, yielding 0.35 ± 0.06 mg/kg BW for Ostarine and 41 ± 8 mg/kg BW for testosterone [22, 23]. Twelve weeks after Orx, all rats underwent bilateral tibia osteotomy with plate osteosynthesis; the data on bone healing have been published previously [22].

Eighteen weeks after orchiectomy, the rats were decapitated under CO_2_ anesthesia, and both femora and fourth lumbar vertebrae (L4) were collected and stored at −20 °C for further analyses. The sixth lumbar vertebral body (L6) was stored at −80 °C for gene expression analysis. Blood samples were collected and the weight of the prostate, heart, liver, kidneys, and spleen was recorded. During the experiments, losses of animals occurred due to complications involved in anesthesia and postoperative pain therapy [22]. The following numbers of animals could be analyzed in each group: Non-Orx: 12, Orx: 8, Ostarine Therapy: 7, Test. Therapy: 5, Ostarine Proph.: 9, and Test. Proph.: 9 [22].

### In vivo quantitative computed tomography (pQCT)

The in vivo pQCT of L4 was performed under isoflurane anesthesia with five rats per group using the pQCT device (XCT Research SA, Stratec Medizintechnik GmbH, Pforzheim, Germany), as described in a previous study [27]. Animals were scanned with the following parameters: 90 mm measurement diameter, 0.2 mm voxel size, 90 s scan time, 0.3 mA anode current, 50 kV high voltage, 180 projections, and 1° angle between detectors. L4 was scanned at week 12 and 18 after Orx. The total bone mineral density (total BMD, mg/cm^3^) was evaluated using XCT-6.20 C software (Stratec Medizintechnik GmbH, Pforzheim, Germany).

### Biomechanical assessment

The biomechanical assessment of bone was performed using a Zwick device (type 145 660 Z020/TND, Zwick, Ulm, Germany) [28, 29]. Samples were thawed and kept moist using saline during biomechanical assessment. For L4, a compression test was performed as described previously [28]. L4 was fixed with the cranial end plate on the aluminum base. The surface of the stamp corresponded to the shape of the caudal vertebral body end plate. The compression load was applied at the caudal end plate along the cranio-caudal axis of L4. The femur was analyzed via a three-point bending test [29]. The femoral head was placed on the base and in a 4-mm deepening made in the aluminum base and loaded on the trochanteric region. The motion of the stamps occurred at 5 mm/min, and the test was stopped when the force declined by at least 10 N for lumbar vertebral body. The test of femur strength was conducted until it was broken. The results were recorded using test Xpert software (Zwick). The maximum load (Fmax, N) and stiffness (S, N/mm) were calculated using Excel (MS Office 2010).

### Micro-computed tomography (micro-CT)

The bone structure of right femur and L4 was analyzed using Quantum FX micro-CT (Caliper Life Sciences, Hopkinton, MA, USA) at 70 kVp and 200 μA with a 2-min exposure time, 360° rotation, 3600 projections, 20 × 20 mm^2^ field of view, 512-pixel matrix, and 40-μm resolution [30]. A phantom with five known mineral densities was included in each scan as an internal control. 3DOsteo analyze software (developed in our laboratory) was used to calculate the bone parameters according to the method of the American Society for Bone and Mineral Research (ASBMR; [31]. A later version, Scry v6.0 software (Kuchel & Sautter UG, Bad Teinach-Zavelstein, Germany), is commercially available [32]. The body of L4 and the femoral head were taken for a three-dimensional analysis (3D) of bone mineral density (BMD, g/cm^3^), bone volume (BV, mm^3^), bone volume fraction (BV/TV, %), cortical density (Ct.BMD, g/cm^3^), and cortical volume (Ct. V, mm^3^). Trabecular BMD and volume were also analyzed and didn´t showed any significant difference between the treatment groups for both L4 and femur (data not shown).

Furthermore, bone structures were assessed using two-dimensional analysis (2D) with the help of MetaMorph Basic Acquisition Software (Leica Mikrosysteme Vertrieb, Wetzlar, Germany) [33]. L4 and the femoral head were cut on the sagittal plane of the 3D images, as shown in Fig. 3. Three central images were used in the 2D analysis. In L4, the growth plate was excluded from the analysis. In the femur, the measured area was between the epiphyseal line of the femoral head and the intertrochanteric line (5 mm distally, including the head without the epiphysis, neck, and trochanteric region). The trabecular nodes (Tb.Nd), trabecular width (Tb.Wi, mm), and trabecular density (Tb.Dn, %) were recorded as described previously [30].

### Ashing

To investigate the organic and inorganic weights, the left femora and L4 were weighed and then heated to 750 °C for 120 min [18]. After ashing, the bones were weighed repeatedly, and the mineral content (ash weight) was calculated relative to the wet weight of each bone (%). Bone ash (50 ± 0.6 mg) was taken to measure the phosphate, magnesium, and calcium content. Phosphate content was assessed using a colorimetric method (spectral photometer DM4, Zeiss, Germany), while the amounts of magnesium and calcium were determined by an absorption spectrometer (4100, PerkinElmer, Waltham, MA, USA) according to the CEN 2002 criteria [34]. Data are shown relative to the sample weight (50 ± 0.6 mg).

### Gene expression analysis

Frozen samples of L6 were homogenized using a micro-dismembrator (Sartorius, Germany). Homogenized bone samples (50 mg; *n* = 5/group) were incubated with 500 μl TRIzol (Thermo Fischer Scientific, WA, USA) for 5 min at room temperature and further processed according to the manufacture’s protocol, using the phenol/chloroform purification of RNA (Thermo Fischer Scientific). Then, RNA was dissolved in 20 μL H_2_O and measured using a DeNovix DS-11 FX+ System (DeNovix, NC, USA).

Reverse transcription was performed with 1000 ng of total RNA using an M-MLV Reverse Transcription Kit according to manufacturer’s instructions (Promega, WI, USA). Quantitative realtime PCR (QRT-PCR) was performed on a CFX96 real-time PCR detection system (Biorad, CA, USA), using SYBR Green (Biorad, CA, USA) as a detection marker.

The gene expression levels of alkaline phosphatase (ALP) (Qiagen, Hilden, Germany, ready to use primers: Cat. no: QT00190680), androgen receptor (AR) (Qiagen, Cat. no: QT 00184394), estrogen receptor α (ERα) (Qiagen, Cat. no: QT01595013), estrogen receptor ß (ERβ) (Qiagen, Cat. no: QT00190113), receptor activator of nuclear factor κB ligand (RANKL) (Qiagen Cat. no: QT00195125), osteocalcin (OC) (Qiagen Cat. no: QT01084573), and osteoprotegerin (OPG) (Qiagen, Cat. no: QT00177170) were measured in triplicate and analyzed via the 2^-ΔΔ*C*T^-method [35], using β-2-microglobulin (Qiagen, Cat. no: QT00176295) as a housekeeping gene.

### Serum analyses

The enzyme immune assay RatLaps CTX-I (AC-06F1, Immunodiagnostic Systems Holdings, Boldon Colliery, UK) was used to measure β-crosslap levels (Col1 degradation product) in serum samples. Osteocalcin levels were measured by the enzyme immune assay Rat-Mid™ Osteocalcin EIA (AC-12F1, Immunodiagnostic Systems Holdings). The analyses were performed according to the manufacturer’s instructions.

Analyses of ALP, magnesium, calcium and phosphorus were performed at the Department of Clinical Chemistry, University Medical Center, Goettingen, Germany, using Abbott’s Architect C16000 analyzer (Abbott, Wiesbaden, Germany) according to the manufacturer’s instructions. Then, ALP activity was measured via the para-nitrophenyl phosphate method at 404 nm (Ref: 7D55-20), calcium content was analyzed via Arsenazo III dye at 660 nm (Ref: 7D61-29), phosphorus amount was analyzed after the reaction of inorganic phosphate with ammonium molybdate at 340 nm (Ref: 7D71-20), and magnesium was quantified using Arsenazo dye at 572 nm (Ref: 7D70-20) (Architect®/Aeroset®, Abbott).

### Statistical analyses

The impact of the treatments was evaluated via one-way ANOVA, and differences between the treatment groups were analyzed via a Tukey–Kramer post-hoc test (GraphPad Prism 5, La Jolla, CA, USA). *P*-values < 0.05 were considered significant. Data are presented as mean values and standard deviations (SDs).

## Results

### Animal model

Food intake did not differ significantly between the treatment groups [22]. No significant differences were observed in the starting body weights of the animal groups (Table 1). Over the entire experiment, the average body weight of the Non-Orx group was significantly higher than those of the other groups. All Orx groups showed a significant reduction in prostate weight as compared to the Non-Orx animals. Ostarine prophylaxis treatment significantly increased prostate weight as compared to the Orx group and both testosterone groups. Neither Orx nor ostarine and testosterone treatments affected the weight of the liver, heart, kidney and spleen (Table 1).

**Table 1:** Starting and average bodyweight, organ weight at the end of the study and in vivo pQCT data of L4

Group	Non-Orx		Orx		Ostarine Therapy		Ostarine Proph.		Test. Therapy		Test. Proph.	
	Mean	SD	Mean	SD	Mean	SD	Mean	SD	Mean	SD	Mean	SD
Starting body weight [g]^#^	717.5	70.5	713.3	75.3	687.9	81.7	715.8	71.2	680.2	74.3	703.4	82.3
Average body weight [g]^§^	730.2	29.6	669.5^a^	34.9	667.1^a^	33.5	683.7^a^	30.3	679.6^a^	25.3	663.7^a^	31.9
Prostate weight [g]^#§^	1.22	0.30	0.18^a,d^	0.07	0.39^a^	0.08	0.62^a,b,e,f^	0.13	0.19^a^	0.07	0.33^a^	0.13
Heart weight [g]	1.75	0.22	1.68	0.15	1.84	0.32	1.84	0.31	1.70	0.31	1.65	0.17
Liver weight [g]	23.1	3.9	19.7	2.4	20.5	1.9	20.7	5.2	20.1	0.5	20.8	2.9
Kidney weight [g]	4.0	0.6	3.5	0.5	3.9	0.6	4.2	0.5	3.3	0.2	3.5	0.6
Spleen weight [g]	1.40	0.23	1.46	0.15	1.35	0.28	1.55	0.27	1.33	0.14	1.58	0.44
Total BMD [mg/ccm] 12 weeks	535.0	46.53	476.2^a^	35.49	489.4	37.06	557.7^b,c^	50.23	493.3^d^	40.0	529.9^b^	49.54
Total BMD [mg/ccm] 18 weeks	475.3	32.38	430.2^a^	38.16	472.6	50.80	526.8^a,b,c^	46.11	465.5^d^	39.9	485.3^b^	23.91

^a^Differs from Non-Orx, ^b^differs from Orx, ^c^differs from Ostarine Therapy group, ^d^differs from Ostarine Proph. group, ^e^differs from Test. Therapy group, ^f^differs from Test. Proph. Group, (*p* > 0.05, Tukey-test)

^#^Published in [23]

^§^Published in [22]

### Bone analyses

#### In vivo pQCT of L4

Twelve weeks after Orx, total BMD was significantly lower in the Orx group as compared to the Non-Orx group, whereas ostarine and testosterone prophylaxis treatments prevented this bone loss and showed increased total BMD as compared with the Orx, Ostarine Therapy, and Test. Therapy groups (Table 1).

At the end of the experiment (week 18), the total BMD of Orx animals was lower as compared to those of the Non-Orx, Ostarine Proph., and Test. Proph. groups (Table 1). In ostarine prophylaxis animals, BMD was higher as compared to the ostarine therapy group, while testosterone-therapy animals had lower BMD as compared to ostarine-prophylaxis animals.

#### Micro-CT Analyses (3D and 2D)

**Fig. 2:**
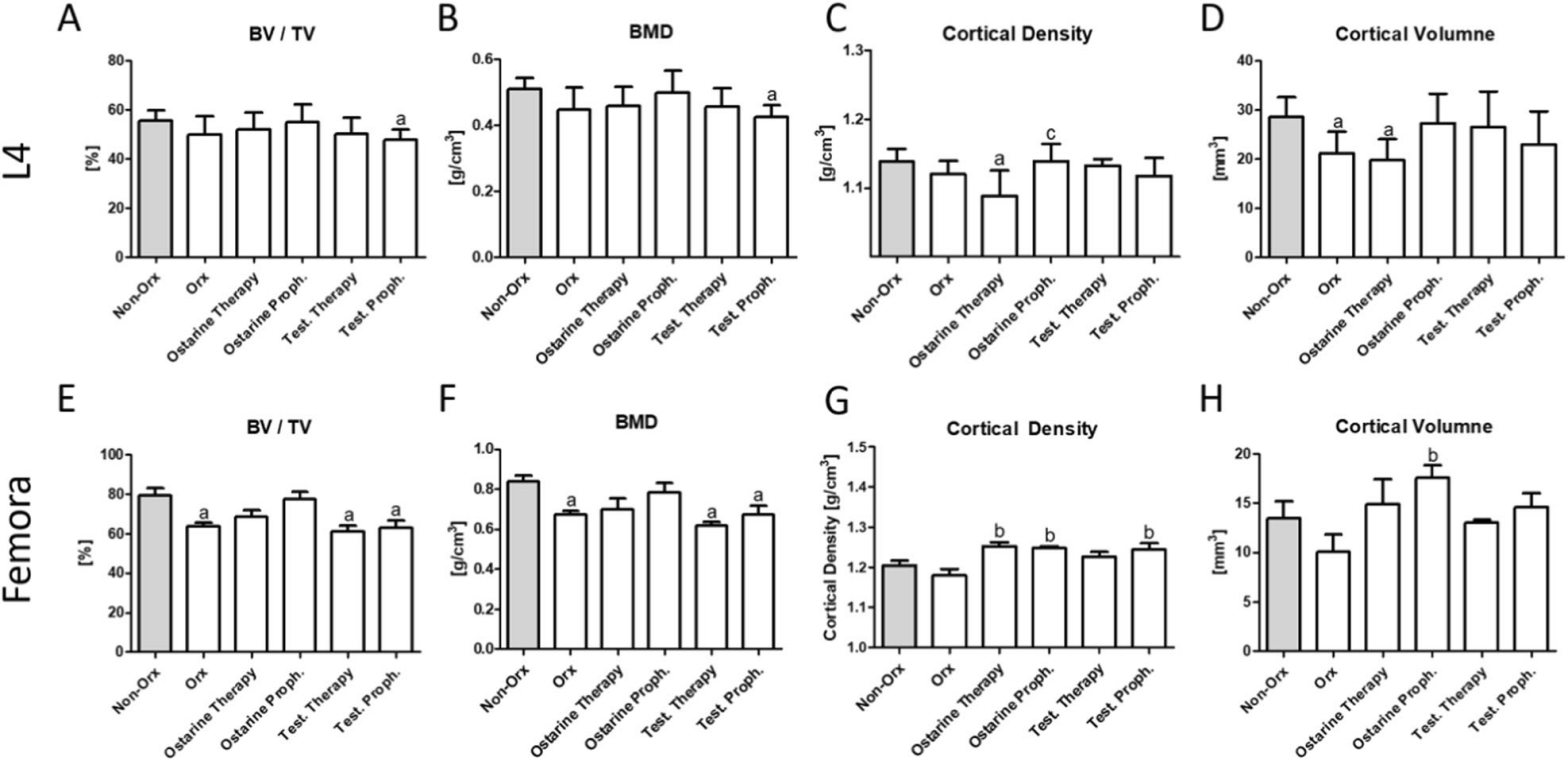
3D micro-CT analysis. 3D analysis of L4 (**A**–**D**) and femur (**E**–**H**) performed after 18 weeks in Non-Orx rats and Orx rats either untreated or treated with ostarine or testosterone. a: differs from Non Orx; b: differs from Orx, c: differs from ostarine Therapy group (*p* < 0.05, Tukey-test)

The 3D analysis of L4 revealed that BV/TV and BMD were lower in the Test. Proph. group than in the Non-Orx group, whereas the differences between the other groups were not significant (Fig. 2A, B). Surprisingly, a decrease in Ct. BMD was detected in the Ostarine Therapy group as compared to Non-Orx and Ostarine Proph. groups (Fig. 2C). Also, Ct.V was reduced in the Orx and Ostarine Therapy groups as compared to the Non-Orx animals (Fig. 2D).

In the femur, lower BV/TV and BMD were revealed in the Orx, Test. Therapy, and Test. Proph. groups as compared to the Non-Orx animals (Fig. 2E, F). A significantly higher Ct.BMD value was detected in the Ostarine Proph. and Test. Proph. groups than in the Orx group (Fig. 2G), whereas Ct.V was higher solely in the Ostarine Proph. group (Fig. 2H).

**Fig. 3:**
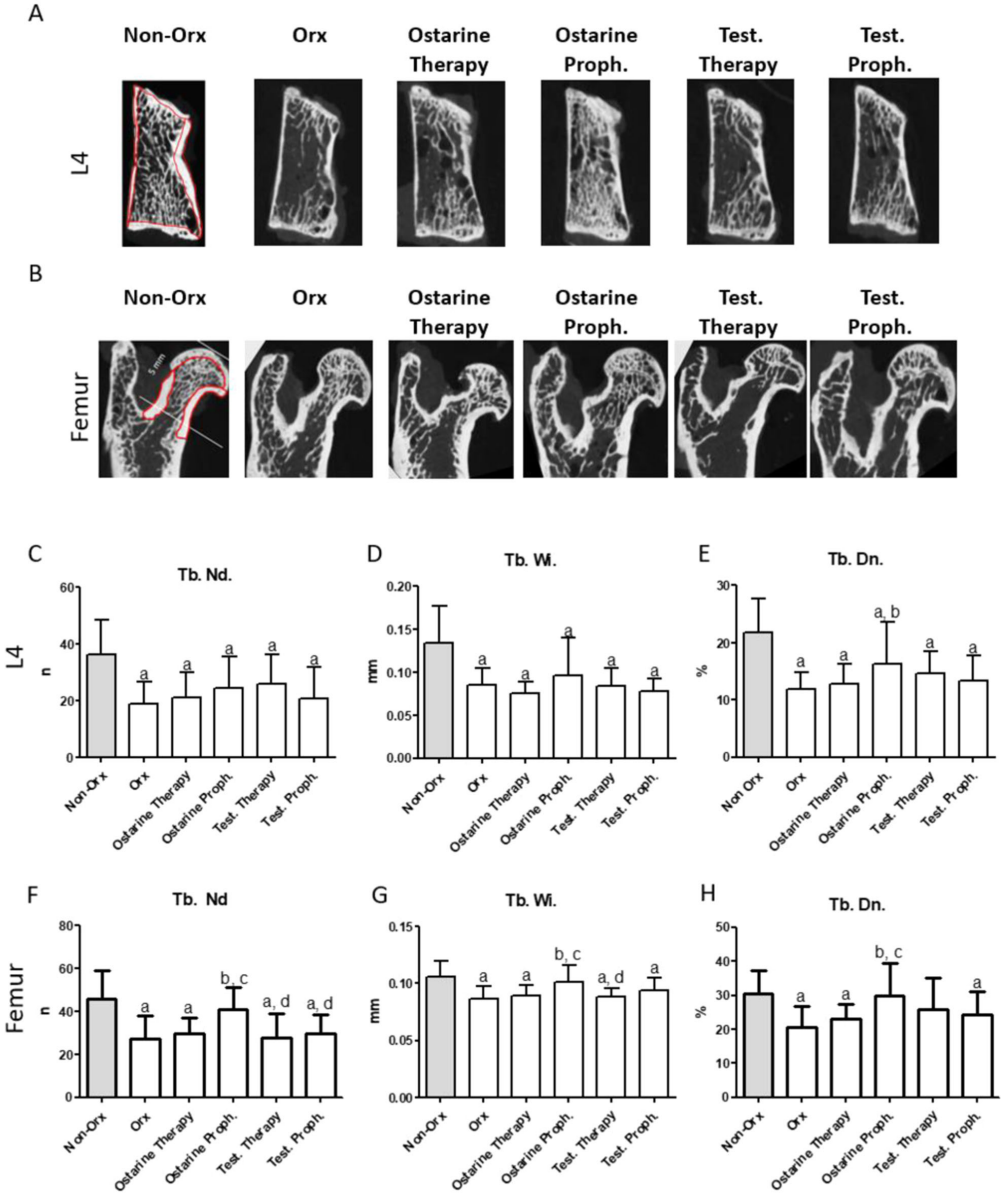
Representative 2D images of L4 and the femur (**A**, **B**). The measurements areas of cortical and trabecular bone in femur and L4 are surrounded by red lines for the Non-Orx groups. 2D analysis of L4 (**C**–**E**) and femur (**F**–**H**) performed 18 weeks after Orx in Non Orx rats and Orx rats either untreated or treated with ostarine or testosterone. a: differs from Non Orx; b: differs from Orx; c: differs from Ostarine Therapy group; d:differs from Ostarine Proph. Group, (*p* < 0.05, Tukey–test)

Representative 2D images of L4 and the femur are shown in Fig. 3A, B. The measurements areas of cortical and trabecular bone in femur and L4 are surrounded by red lines for the Non-Orx groups. In L4, significant reductions in Tb.Nd, Tb.Wi, and Tb.Dn were detected in all treatment groups as compared to the Non-Orx group via the 2D analysis (Fig. 3C–E). Ostarine prophylaxis treatment led to a significant increase in Tb.Dn as compared to the Orx group (Fig. 3E). In the femur, all trabecular parameters were lower in the Orx group than in the Non-Orx group (Fig. 3F–H). Ostarine Proph. improved these parameters, whereas Ostarine Therapy and Test. Proph. showed no effect. In the Test. Therapy group, Tb. Dn was not different from the other groups (Fig. 3H).

#### Biomechanical analysis

**Fig. 4:**
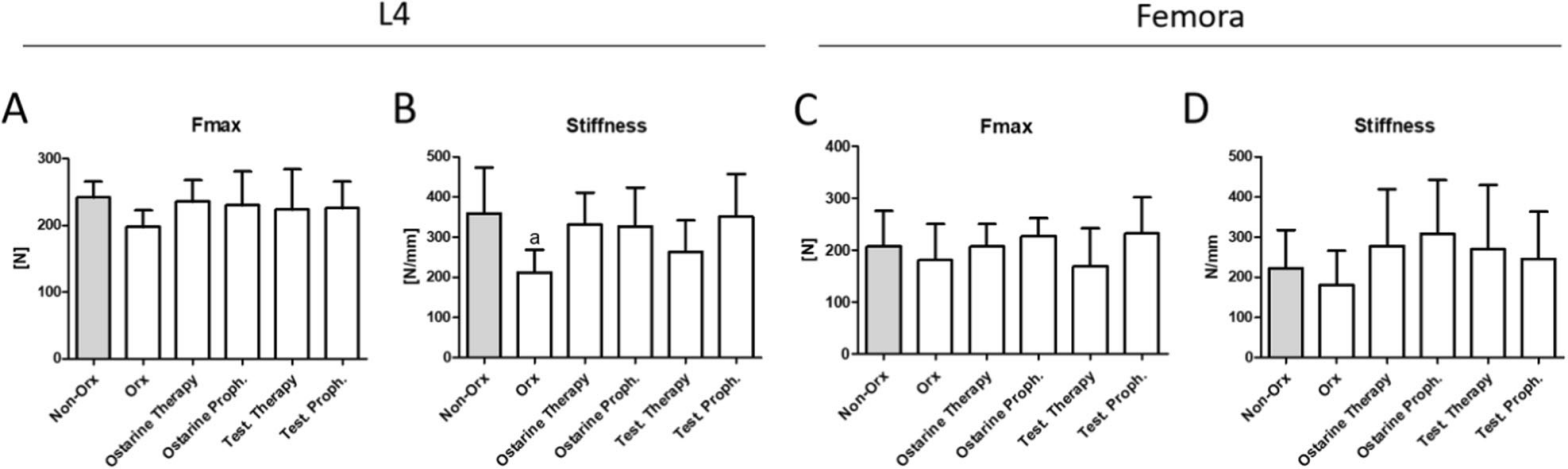
Biomechanical analysis of L4 (**A**, **B**) and femur (**C**, **D**) performed 18 weeks after Orx in Non-Orx rats and Orx rats either untreated or treated with ostarine or testosterone. a: differs from Non-Orx (*p* < 0.05, Tukey–test)

In L4, no significant differences in Fmax were observed between the groups (Fig. 4A). Stiffness was significantly lower in the Orx group than in the Non-Orx group, while all treatments could restore the Non-Orx level (Fig. 4B). In the femur, the differences between the treatment groups in terms of biomechanical parameters were not significant (Fig. 4C, D).

#### Serum analysis

Ostarine Therapy treatment caused a significant reduction in serum OC levels as compared to the Orx, Test. Therapy and Test. Proph groups. The OC level was also lower in the Ostarine Proph. group than in the Orx and Test. Therapy groups. ALP activity and CTX-I, calcium, and magnesium levels did not differ between the groups. A significantly higher concentration of phosphorus was detected for the Test. Proph. and Ostarine Proph. treatments as compared to Orx rats (Table 2).

**Table 2:** Serum analysis performed 18 weeks after Orx in Non-Orx rats and Orx rats either untreated or treated with ostarine or testosterone

Group	Non-Orx		Orx		Ostarine Therapy		Ostarine Proph.		Test Therapy		Test Proph.	
	Mean	SD	Mean	SD	Mean	SD	Mean	SD	Mean	SD	Mean	SD
OC [ng/ml]^§^	174.5	23.9	207.6	54.3	123.4^b,c,d^	26.8	138.4^b,c^	26.4	200.4	32.5	183.2	31.6
CTX-I [ng/ml]^§^	16.7	3.32	18.1	4.2	16.6	1.4	16.9	4.2	18.8	2.6	19.6	2.1
ALP [U/l]^#^	182.2	53.8	133.7	38.0	194.2	43.3	181.6	70.2	147.2	34.9	150.6	27.9
Ca [mmol/l]^#^	2.17	0.20	2.06	0.16	2.03	0.11	2.16	0.16	2.21	0.22	2.28	0.12
Mg [mmol/l]^#^	0.74	0.10	0.68	0.07	0.70	0.05	0.74	0.07	0.75	0.08	0.77	0.06
Phosphorus [mmol/l]^#^	1.84	0.26	1.56	0.19	1.79	0.22	2.03^b^	0.27	1.68	0.24	1.99^b^	0.25

^b^Differs from Or

^c^Differs from Test. Therapy group

^d^Differs from Test. Proph. Group (*p* < 0.05, Tukey–test)

^#^Published in [23]

^§^Published in [22]

#### Mineral content analysis

In L4, calcium, magnesium, and phosphate content did not differ between the groups (Table 3). Test. Proph. treatment decreased the calcium-phosphate quotient as compared to the Non-Orx and Orx groups. Mineral content was lower in the Orx group as compared to the Non-Orx animals, whereas Ostarine Proph. treatment significantly increased it as compared to the Orx group.

**Table 3:** Mineral content of L4 and femora performed 18 weeks after Orx in Non-Orx rats and Orx rats either untreated or treated with ostarine or testosterone

Group	Non-Orx		Orx		Ostarine Therapy		Ostarine Proph.		Test Therapy		Test Proph.	
	Mean	SD	Mean	SD	Mean	SD	Mean	SD	Mean	SD	Mean	SD
L4												
Calcium [%]	36.41	1.19	35.88	0.53	36.20	0.49	36.09	0.84	36.31	0.75	36.00	0.27
Magnesium [%]	0.72	0.03	0.70	0.03	0.70	0.04	0.69	0.03	0.67	0.04	0.70	0.02
Phosphate [%]	63.6	2.2	62.6	1.5	63.7	1.4	64.2	1.5	64.2	1.8	64.4	0.57
Ca/PO_4_	0.77	0.02	0.78	0.01	0.77	0.01	0.76	0.01	0.77	0.01	0.75^a,b^	0.004
Mineral content [%]	31.3	1.7	27.8^a^	2.9	30.5	3.0	31.4^b^	1.9	28.4	1.8	29.1	1.2
Femora												
Calcium [%]	35.28	0.89	35.13	1.39	35.69	0.67	35.70	0.49	35.21	0.62	35.88	0.39
Magnesium [%]	0.52	0.04	0.52	0.05	0.54	0.03	0.56	0.04	0.56	0.03	0.63^a,b,c,d,e^	0.01
Phosphate [%]	54.9	1.5	54.6	2.3	55.3	1.4	56.1	0.6	54.8	1.2	56.0	1.0
Ca/PO_4_	1.52	0.01	1.53	0.02	1.53	0.02	1.51	0.01	1.52	0.02	1.52	0.01
Mineral content [%]	45.8	6.3	39.7^a^	2.7	41.3	1.9	36.6^a^	3.7	39.4	3.9	40.6	1.9

^a^Differs from Non-Orx, ^b^differs from Orx, ^c^differs from Ostarine Therapy group, ^d^differs from Ostarine Proph. group, ^e^differs from Test. Therapy group, (*p* > 0.05, Tukey-test)

In the femora, Test. Proph. treatment led to increased magnesium content as compared to all other groups, while calcium and phosphate levels remain unaffected. Mineral content was lower in the Orx and Ostarine Proph. groups as compared to the Non-Orx animals (Table 3).

#### Gene expression analysis in L6

**Fig. 5:**
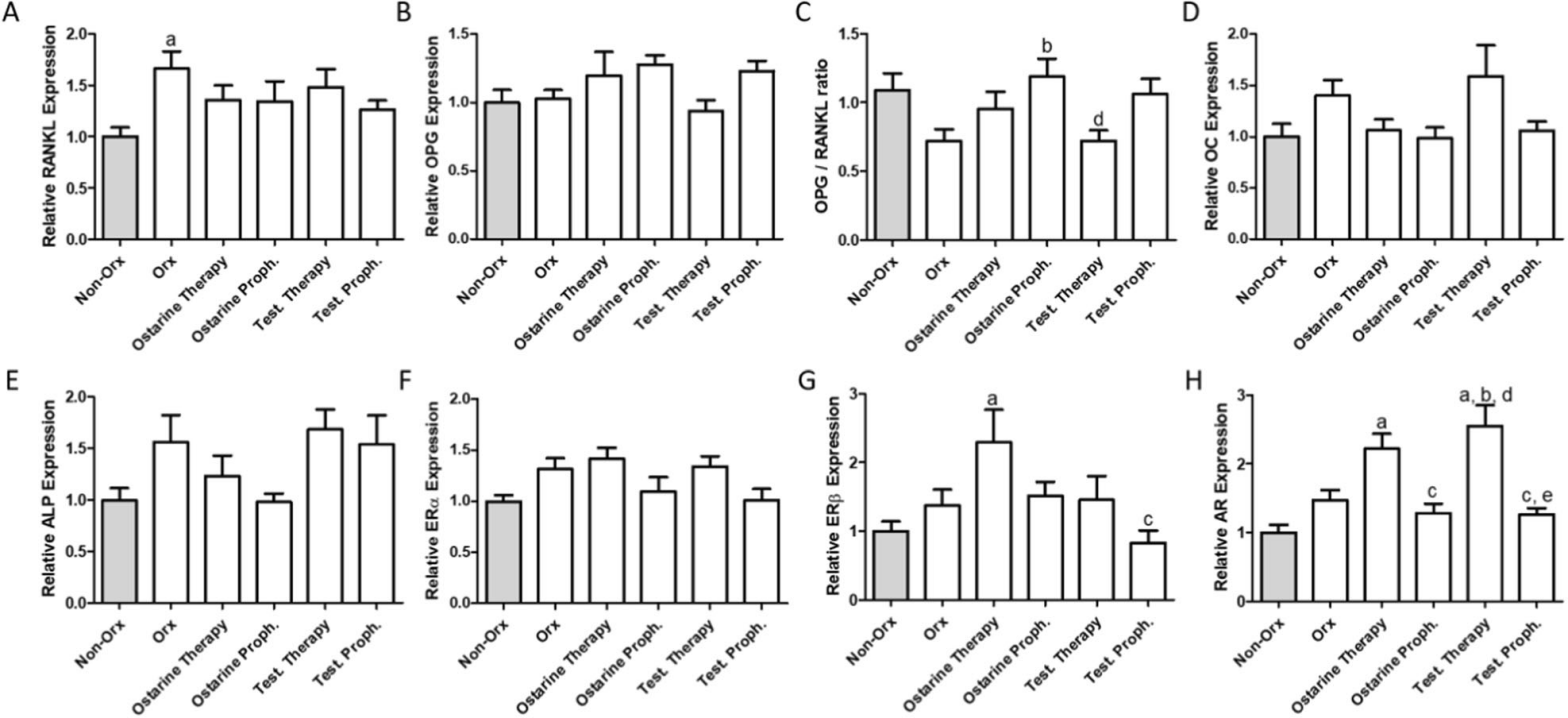
Gene expression analysis in L6 performed 18 weeks after Orx in Non-Orx rats and Orx rats either untreated or treated with ostarine or testosterone. Relative expression of RANKL (**A**), OPG (**B**), OPG/RANKL ratio (**C**), OC (**D**), ALP (**E**), ERα (**F**), ERβ (**G**) and AR (**H**) were analyzed. a: differs from Non-Orx group; b: differs from Orx group, c: differs from Ostarine Therapy group, d: differs from Ostarine Proph. Group, e: differs from Test. Therapy group, f: differs from Test. Proph. group (*p* < 0.05, Tukey-test)

RANKL expression was higher in the Orx group as compared to Non-Orx animals. The differences between the other groups were not significant (Fig. 5A). The expression levels of OPG, ALP, Erα, and osteocalcin did not differ between groups (Fig. 5B, D–F), but the OPG/RANKL quotient was significantly increased in the Ostarine Proph. group as compared to the Orx and Test Therapy groups (Fig. 5C). Higher mRNA expression of ERβ was detected in the Ostarine Therapy group, which represented a significant difference as compared to the Test. Proph. and Non-Orx groups (Fig. 5G). AR expression was higher in the Ostarine Therapy and Test. Therapy groups than in the Non-Orx, Ostarine Proph., and Test. Proph. groups. In the Test. Therapy group, this value was also significantly different from that in the Orx group (Fig. 5H).

## Discussion

In our study, we present ostarine’s effects on structural and chemical parameters, biomechanical stability, and gene expression at two skeletal sites, the lumbar spine and femur. This study provides new insights into the important topic of male osteoporosis. In the present study, Ostarine Proph. treatment prevented bone deterioration in male rats after Orx by maintaining the BMD of L4 at a higher level than in Orx rats, as was determined via in vivo pQCT analysis at weeks 12 and 18 after Orx. After 18 weeks had passed since Orx, this treatment even improved BMD as comparing to Non-Orx rats. The favorable effect of Ostarine Proph. was supported by a detailed micro-CT analysis of the bone structure. Enhanced cortical density and volume were measured via 3D micro-CT analysis in the femur, whereas in both L4 and the femur, trabecular density was higher in this group than in the Orx rats based on 2D micro-CT analysis. In the femur, the effect was more pronounced than in L4, with additionally elevated trabecular thickness and number of nodes in the Ostarine Proph. group. In contrast, mineral content in the Orx rats was maintained under Ostarine Proph. in L4, whereas in the femur, it was at the lower level seen in Orx rats. This could be explained by the heterogenous changes that occur in various skeletal parts during osteoporosis [36–39]. Bone loss during osteoporosis depends on bone localization, and even anti-osteoporotic treatment varies between femora and vertebrae samples [37, 40]. Ostarine applied as a therapeutic treatment for 6 weeks was effective solely in improving the cortical density of the femur. Perhaps, a prolonged treatment could have a stronger effect on osteoporotic bone tissue. The osteoanabolic effects of SARMs have been previously described in the literature; e.g., studies on SARM drugs such as S-4 or LGD-3303 have shown their positive effects on bone tissue. Kearbey et al. [41, 42] showed that S-4 treatment maintained trabecular BMD, cortical content, and increased bone strength after 120 days of treatment in ovariectomized rats. Vajda et al. [43] used another SARM, LGD-3303, which was orally administered for 14 days in osteopenic female rats, and found increased bone density at cortical and cancellous bone sites [43].

In addition to the osteoanabolic effects of SARMS, inhibitory effects on bone resorption are described. S-4 showed antiresorptive effects by decreasing TRAP-positive multinucleated cells in an in vitro study [42]. Furthermore, beneficial effects of ostarine on muscle structure and vascularization in male rats have been observed in previous studies [23], which may indirectly improve bone tissue. Ostarine Therapy treatment did not change bone’s structural and chemical parameters, likely having been too short to ameliorate osteoporotic changes in bone. Testosterone treatments showed less effect than ostarine on bone structure and quality in the femur and vertebral body, irrespective of application regime (Proph. or Therapy). The cortical density of the femur was solely enhanced in the Test. Proph. group. In contrast, these testosterone treatments had a positive effect on bone healing for osteotomized tibiae, which was stronger than the effect of ostarine treatments [22]. This can be explained by the aromatization of testosterone to estrogen, which is absent in ostarine [17], and testosterone could act not only through ARs but also through ERs [22]. Apparently, the dosage and oral application of testosterone propionate were sufficient to improve bone healing in male rats [22] but failed to affect non-osteotomized femur and vertebral body in the present study. This likely occurred due to the differences in metabolic processes at the fracture site and in intact bone. Bone turnover during fracture healing is elevated [44], whereas in uninjured osteoporotic bone, the remodeling processes slow down with time [45].

Further analysis of bone minerals showed that testosterone prophylaxis treatment increased the magnesium levels in femur samples and decreased the Ca/PO_4_ ratio in L4 as compared to Non-Orx and Orx animals. In serum, the phosphorus level after both prophylaxis treatments (Ostarine and Testosterone) was significantly increased as compared to that in Orx animals. Nevertheless, the significance of calcium, magnesium, and phosphorus in bone and serum is limited due to the kinetics of bone metabolism, their ubiquitous occurrence, and their diverse involvements in general metabolic processes [46, 47]. For instance, increased parathyroid activity can lead to increased serum magnesium levels and lower calcium Ca/PO_4_ ratios [48]. Therefore, serum was also analyzed for alkaline phosphatase (ALP), osteocalcin (OC), and collagen 1 degradation product (CTX-I). These peptides are biomarkers of bone turnover and can provide insights into the remodeling processes of bone. ALP and OC are markers of bone formation and products of active osteoblast metabolism, while CTX-I is a product of active osteoclasts and, therefore, a marker of bone resorption [49]. OC was reduced in both ostarine groups as compared to the Orx and Testosterone Therapy groups, suggesting reduced bone turnover after ostarine treatment. These data support antiresorptive activity on the part of ostarine, which was also reported after the treatment of orchiectomized rats with another SARM, andarine [50]. ALP and CTX-I levels did not change between the groups, implying low protein degradation in terms of, e.g., collagen I.

The expression analysis of bone genes in L6 showed increased RANKL gene expression in the Orx group, which is in line with the literature and can be explained by increased osteoclast activity [51]. After orchiectomy, the bone mass and the absolute number of osteoclasts are reduced, although the activity of existing osteoclasts is increased [52]. Nevertheless, none of the tested treatments showed a significant effect on RANKL expression. For OPG, no difference in gene expression was detected, whereas OPG/RANKL quotient was increased by ostarine prophylaxis treatment as compared to Orx rats, confirming the positive structural changes in bone observed in this study. ALP showed no significant difference in gene expression, which is in line with the non-significant changes in ALP observed in the serum analysis. OC expression did not differ significantly between the groups, but in the serum, it was elevated. The level of protein synthesis does not always correspond with mRNA expression [53]. These parameters were measured at the end of the study, and the dynamics of their expression remains unknown. No changes were observed for ERα expression, while ERβ expression was significantly increased after ostarine therapy treatment as compared to Non-Orx animals. ERβ plays an important role in regulating cellular mechanotransduction events in osteoblasts, e.g., in ERK phosphorylation and MAPK pathway activation, as well as being increased in COX-2 expression [54]. On the other hand, AR expression was only affected by both therapy treatments, while prophylaxis treatments did not change AR expression significantly. AR expression has been found in whole bone marrow obtained from mice, and AR is also widely expressed in human bone and bone marrow [55]. Because therapy treatments solely influenced AR expression, this effect seems to be time dependent and was negated in prophylaxis treatments.

Despite significant improvements in bone structure under ostarine treatments, the biomechanical properties of bone were only slightly changed. In femora no significant biomechanical changes were observed, while the reduced stiffness in L4 due to Orx treatment could be rescued by all tested treatments. Similar results were observed in a female rat model, in which the bone stiffness of femora was not affected but the significant structural improvements in bone tissue were measured under ostarine treatment [18]. Ostarine treatment should likely be combined with other bone-sparing substances, e.g., SERMs [56]. In previous studies, SARMs (S-101479 and ostarine), predominantly bone-anabolic substances, when applied in combination with the SERM raloxifene, known as an antiresorptive drug, improved bone parameters to a greater extent than single compounds in female and male rat models [56, 57].

At the end of experiment, Orx was checked visually via the absence of testis and confirmed based on an atrophied prostate. The detrimental effect on the part of Orx on bone tissue was confirmed by reduced bone structural parameters, as well as reduced biomechanical properties, like a reduction of stiffness in L4, while all treatments maintained the values at the level of the Non-Orx group. Orx also caused a significant decrease of BW, while the ostarine and testosterone treatments did not change this effect. Food intake did not differ between the groups in this study [22] as well as weight of inner organs. As previously reported, BW did not directly reflect food intake [58], while metabolic changes [59] and bone and muscle loss [60] may reduce BW in Orx rats. Furthermore, the strong reduction in prostate weight observed in Orx rats was diminished by the Ostarine Proph. treatment. Similar results were obtained for the SARM S-4, which affected prostate weight after Orx surgery [50]. This indicates that the effect of ostarine applied for a prolonged time as a prophylaxis treatment reduced its selectivity for the musculoskeletal system and also affected the sex organs. Furthermore, prostate weight was not affected by oral treatment with testosterone, which can be explained by its limited bioavailability when using this administration route, which was chosen based on previous studies showing favorable effects on bone tissue [11, 26, 61]. Other studies have applied testosterone at higher concentrations (100 mg/kg BW) and used injections instead of oral intake [62, 63]. The clinical application of testosterone as a hormone replacement therapy is limited due to its side effects [12]. However, testosterone treatments are usually included as controls in experimental designs, and therefore, we suggest higher doses and injections instead of oral administration in future studies.

In sum, Ostarine Proph. treatment showed positive effects in terms of preventing osteoporotic changes in cortical and trabecular bone. Ostarine Therapy treatment had less effect, solely improving the cortical density of femur. Thus, in monotherapy, the effect of ostarine does not appear to be sufficient to significantly reduce the development and progression of osteoporosis. Combination therapies of SARM and SERM could be considered in future studies, e.g., a combination of ostarine and raloxifene [23]. By applying this combination therapy, the anabolic influence on musculoskeletal tissue is maintained, whereas the androgenic effect on the prostate is reduced [23, 56].

Test. Proph. treatment positively affected only cortical density in the femur, whereas Test. Therapy did not change any bony parameters, likely due to the low dosage and oral route of administration. In future studies, higher doses and administration via injection should be considered when applying testosterone treatments. In general, prophylaxis treatments showed stronger effects compared to therapy treatments. In order to exclude the effect of treatment duration (18 weeks for prophylaxis treatment compared to 6 weeks for therapy treatment), further studies could be performed with the same administration times.

Concluding, Ostarine Proph. treatment could be further investigated as a preventative treatment for osteoporosis in orchiectomized males, but the androgenic effect on the prostate should be taken into consideration, and combination therapies with other agents could be considered.
